# Prognostic and predictive factors for Taiwanese patients with advanced biliary tract cancer undergoing frontline chemotherapy with gemcitabine and cisplatin: a real-world experience

**DOI:** 10.1186/s12885-020-06914-1

**Published:** 2020-05-14

**Authors:** Chiao-En Wu, Wen-Chi Chou, Chia-Hsun Hsieh, John Wen-Cheng Chang, Cheng-Yu Lin, Chun-Nan Yeh, Jen-Shi Chen

**Affiliations:** 1 Division of Haematology-Oncology, Department of Internal Medicine, Chang Gung Memorial Hospital at Linkou, Chang Gung University College of Medicine, 5, Fu-Hsing Street, Kwei-Shan Taoyuan, Taiwan; 2 Department of Gastroenterology, Chang Gung Memorial Hospital at Linkou, Chang Gung University College of Medicine, 5, Fu-Hsing Street, Taoyuan, Kwei-Shan Taiwan; 3 Department of General Surgery, Chang Gung Memorial Hospital at Linkou, Chang Gung University College of Medicine, 5, Fu-Hsing Street, Kwei-Shan Taoyuan, Taiwan

**Keywords:** Biliary tract cancer, Chemotherapy, Gemcitabine, Cisplatin, Prognostic factor

## Abstract

**Background:**

Chemotherapy with gemcitabine and cisplatin has been the standard of care in first-line chemotherapy for advanced biliary tract cancer (BTC) since the trial ABC-02 was published in 2010. We aimed to investigate the prognostic and predictive factors of this regimen in a cohort of Taiwanese patients with advanced BTC.

**Methods:**

A total of 118 patients with histologically confirmed BTC treated at Chang Gung Memorial Hospital at Linkou from 2012 to 2017 were retrospectively reviewed.

**Results:**

The median progression-free survival (PFS) and overall survival (OS) were 3.6 months and 8.4 months, respectively. In the multivariate analysis, neutrophil to lymphocyte ratio (NLR) > 7.45, biliary drainage requiring both percutaneous transhepatic cholangiography drainage (PTCD) and internal stenting, and tumor responses with progressive diseases and not assessed were independent poor prognostic factors for PFS. Male sex, NLR > 7.45, alkaline phosphatase> 94 U/L, biliary drainage requiring both PTCD and internal stenting, and tumor responses with stable disease, progressive diseases and not assessed were independent poor prognostic factors for OS. Monocyte to lymphocyte ratio (MLR) ≤ 0.28 was the only significant predictive factor for the tumor response. Patients with complete response/partial response had significantly lower MLR than patients with other tumor responses.

**Conclusion:**

We identified three important prognostic factors, namely tumor response, NLR, and biliary drainage requiring both PTCD and internal stenting for both PFS and OS. MLR was the only significant predictive factor for the tumor response. These findings could provide physicians with more information to justify the clinical outcomes in patients with advanced BTC in real-world practice.

## Background

Biliary tract cancers (BTCs) are a group of relatively rare cancers arising from the epithelium of the biliary tract. Their incidence keeps increasing worldwide [1–3]. BTCs including intrahepatic cholangiocarcinoma (iCCA), common bile duct cancer, gallbladder cancer, and ampullary cancer have aggressive biological behaviour, as they are diagnosed at an advanced stage with poor prognosis or high recurrence rate after primary operation [4]. Chemotherapy with gemcitabine and cisplatin has been the standard of care in first-line chemotherapy since the trial ABC-02 was published in 2010 [5]. Some clinical trials have evaluated molecular targeted therapies in combination with chemotherapy and some phase II trials have shown improvement in the patients’ survival outcomes. However, all the completed phase III trials [6–9] and most phase II studies [10–13] did not demonstrate significant improvement in progression-free survival (PFS) and overall survival (OS) [14]. Therefore, chemotherapy is still the standard treatment in advanced BTC.

We previously assessed the efficacy and safety of a chemotherapy regimen with gemcitabine and cisplatin in 30 patients with advanced BTC in a study published in 2012 and showed that this regimen was feasible with manageable toxicity in clinical practice [15]. Currently, this regimen is still the standard of care for advanced BTC and has been reimbursed by Taiwan national health insurance since 2016. Therefore, we aimed to investigate the prognostic and predictive factors of this regimen in a larger cohort of Taiwanese patients with advanced BTC.

## Methods

### Patients

All patients with histologically confirmed BTC treated at the Chang Gung Memorial Hospital (CGMH), Linkou from 2012 to 2017 were retrospectively reviewed. A total of 118 patients with advanced BTC undergoing chemotherapy with gemcitabine and cisplatin were enrolled for further analysis.

### Treatment

The chemotherapy regimen consisted of gemcitabine 1000 mg/m^2^ and cisplatin 30 mg/m^2^ on day 1 and day 8 every 3 weeks according to the treatment guidelines followed at CGMH, Linkou [15]. The dose and the schedule might be adjusted by the physicians according to patients’ clinical status and toxicity from the chemotherapy. The tumor response was evaluated by computed tomography (CT) scan every 3–4 months or as needed.

### Patients’ characteristics and evaluation of outcomes

All patients with advanced BTC treated from 2012 to 2017 were retrospectively reviewed and the patients undergoing gemcitabine and cisplatin as first-line chemotherapy were included in the current study. The patients were followed-up until 31 October 2018. Patients’ characteristics including sex, age, Eastern Cooperative Oncology Group (ECOG) performance status, cancer sites according to the international classification of diseases (10th version), and tumour involvement (primary tumours, regional lymph nodes, and distant metastases) were recorded. The patients with biliary obstruction requiring biliary drainage before chemotherapy were recorded and all the patients should keep drainage lifelong unless surgical intervention could be performed. The patients requiring biliary drainage after starting chemotherapy were not counted in current study as most of them occurred due to disease in progression.

Baseline haemogram and biochemistry including white blood cells, differential counts of white blood cells, platelet count, albumin, total bilirubin, alkaline phosphatase (ALP), alanine aminotransferase, creatinine, carbohydrate antigen 19–9 (CA19–9), and carcinoembryonic antigen (CEA) were recorded. Neutrophil to lymphocyte ratio (NLR), monocyte to lymphocyte ratio (MLR), and platelet to lymphocyte ratio were calculated.

To analyze the NLR, MLR, PLR as the prognostic factors for survivals, recursive partitioning analysis, a statistical method of the survival tree developed by Hothorn, et al. [16] was used to establish an optimal cut-off point that predicts the survivals. However, no significant cut-off value was found for MLR and PLR so the cut-off points of the MLR and PLR were determined by ROC analysis using Youden’s index. The thresholds employed for albumin, ALT, bilirubin, ALP, creatinine, CA19–9, CEA were the limit of their respective normalcy ranges.

The best response including complete response (CR), partial response (PR), stable disease (SD), and progressive disease (PD) were evaluated using the RECIST 1.1 criteria. Patients who experienced rapid deterioration but lacked the images documented before death were recorded as not assessed (N/A). Response rate (RR) was the sum of CR and PR and disease control rate (DCR) was the sum of CR, PR, and SD. The median PFS was defined from the first day of the treatment to the first evidence of disease progression, death, or last follow-up. The median OS was defined from the first day of the treatment to the day of death or last follow-up.

### Statistical analysis

To identify the possible predictive factors, Pearson’s chi-squared test of independence was used for categorical variables. Kruskal-Wallis test, a nonparametric (distribution**-**free) test, was used for the continuous variables. The survival was estimated using the Kaplan-Meier method and comparison of survival was performed by the log-rank test. Univariate and multivariate analyses were performed to evaluate possible prognostic factors. Only the significant prognostic factors were further analysed using the multivariate analysis. IBM SPSS Statistics for Windows (Version 20.0, Chicago, IL, USA) was used for statistical analyses and *P* < 0.05 was considered statistically significant. This study was approved by the institutional review board of Chang Gung Medical Foundation (201901322B0).

## Results

### Patient characteristics

A total of 118 patients with advanced BTC undergoing chemotherapy with gemcitabine and cisplatin as first-line treatment were enrolled in the current study. The mean age was 61.0 years. Sixty patients (50.8%) were female and 58 patients (49.6%) were male. Most of the patients had ECOG performance status≤ 1 (*n* = 102, 86.4%). The clinical features and tumour involvements are summarised in Table 1.

**Table 1:** Patients’ Characteristics and Association with Tumor Response

Characteristics	Total (*N* = 118)	Tumor Response			*P* value
		CR/PR (*N* = 15)	SD (*N* = 41)	PD/NA (*N* = 62)	
Age, median (IQR)	61.0 (14.0)	61.0 (45.0)	60 (15.0)	61.5 (13.0)	.890
**≦**65	74 (62.7)	9 (60.0)	26 (63.4)	39 (62.9)	.972
> 65	44 (37.3)	6 (40.0)	15 (36.6)	23 (37.1)	
Gender					.637
Male	58 (49.2)	7 (46.7)	18 (43.9)	33 (53.2)	
Female	60 (50.8)	8 (53.3)	23 (56.1)	29 (46.8)	
ICD-10 cancer site					.390
C22.1 - ICCA	86 (72.9)	10 (66.7)	33 (80.4)	43 (69.4)	
C23/C24.9 -GB/others	18 (15.3)	2 (13.3)	4 (9.8)	12 (19.4)	
C24.0 – ECCA	9 (7.6)	3 (20.0)	2 (4.9)	4 (6.5)	
C24.1 – Ampullary	5 (4.2)	0	2 (4.9)	3 (4.7)	
Performance status					.254
0/1	102 (86.4)	15 (100.0)	35 (85.4)	52 (83.9)	
2/3	16 (13.6)	0	6 (14.6)	10 (16.1)	
NLR	3.9 (3.4)	3.4 (4.6)	3.3 (3.1)	4.4 (3.2)	.202
**≦**7.45	100 (84.7)	14 (93.3)	35 (85.4)	51 (82.3)	.559
> 7.45	18 (15.3)	1 (6.7)	6 (14.6)	11 (17.7)	
MLR	0.40 (0.32)	0.26 (0.28)	0.29 (0.32)	0.43 (0.31)	.043
**≦**0.28	40 (33.9)	8 (53.3)	19 (46.3)	13 (21.0)	.007
> 0.28	78 (66.1)	7 (46.7)	22 (53.7)	49 (79.0)	
PLR	151.9 (121.2)	173.8 (115.5)	132.7 (114.4)	161.7 (121.7)	.364
**≦**136.4	47 (39.8)	6 (40.0)	22 (53.7)	19 (30.6)	.065
> 136.4	71 (60.2)	9 (60.0)	19 (46.3)	43 (69.4)	
Albumin (g/dL)	3.8 (0.9)	4.0 (1.1)	3.8 (0.8)	3.7 (0.8)	.252
**≦**3.5	33 (31.4)	5 (33.3)	8 (22.9)	20 (36.4)	.399
> 3.5	72 (68.6)	10 (66.7)	27 (77.1)	35 (63.6)	
ALT (U/L)	30.0 (34.0)	30.0 (31.0)	36.0 (56.0)	27.0 (24.0)	.321
**≦**36	68 (58.1)	9 (60.0)	21 (51.2)	38 (62.3)	.532
> 36	49 (41.9)	6 (40.0)	20 (48.8)	23 (37.7)	
Bilirubin (mg/dL)	0.7 (0.8)	0.4 (0.9)	0.6 (0.9)	0.7 (0.9)	.221
**≦**1.3	89 (76.1)	12 (80.0)	31 (75.6)	46 (75.4)	.929
> 1.3	28 (23.9)	3 (20.0)	10 (24.4)	15 (24.6)	
ALP (U/L)	159.5 (168.0)	106.0 (124.5)	159.0 (207.0)	173.0 (147.0)	.108
**≦**94	30 (26.8)	7 (50.0)	11 (29.7)	12 (19.7)	.061
> 94	82 (73.2)	7 (50.0)	26 (70.3)	49 (80.3)	
Creatinine (mg/dL)	0.7 (0.4)	0.7 (0.4)	0.6 (0.3)	0.7 (0.4)	.814
**≦**1.27	115 (97.5)	15 (100.0)	39 (95.1)	61 (98.4)	.470
> 1.27	3 (2.5)	0	2 (4.9)	1 (1.6)	
CA19–9 (U/mL)	282.4 (2808.4)	221.9 (3604.4)	389.9 (1952.9)	260.7 (3096.2)	.621
**≦**37	35 (29.9)	5 (33.3)	7 (17.1)	23 (37.7)	.079
> 37	82 (70.1)	10 (66.7)	34 (82.9)	38 (62.3)	
CEA (ng/mL)	4.3 (15.3)	4.7 (21.2)	3.1 (10.3)	5.6 (19.5)	.259
**≦**5	64 (54.2)	8 (53.3)	26 (63.4)	30 (48.4)	.324
> 5	54 (45.8)	7 (46.7)	15 (36.6)	32 (51.6)	
Biliary drainage					.398
None	88 (74.6)	9 (60.0)	31 (75.7)	48 (77.5)	
Internal stenting	8 (6.8)	3 (20.0)	3 (7.3)	2 (3.2)	
PTCD	19 (16.1)	3 (20.0)	6 (14.6)	10 (16.1)	
Both	3 (2.5)	0	1 (2.4)	2 (3.2)	
Tumor involvement					
Primary tumor					.116
No	8 (6.8)	0	1 (2.4)	7 (11.3)	
Yes	110 (93.2)	15 (100.0)	40 (97.6)	55 (88.7)	
Regional LAP					.585
No	42 (35.6)	7 (46.7)	13 (31.7)	22 (35.5)	
Yes	76 (64.4)	8 (53.3)	28 (68.3)	40 (64.5)	
Lung					.069
No	95 (80.5)	15 (100.0)	34 (82.9)	46 (74.2)	
Yes	23 (19.5)	0	7 (17.1)	16 (25.8)	
Bone					.748
No	105 (89.0)	14 (93.3)	37 (90.2)	54 (87.1)	
Yes	13 (11.0)	1 (6.7)	4 (9.8)	8 (12.9)	
Liver					.465
No	69 (58.5)	10 (66.7)	26 (63.4)	33 (53.2)	
Yes	49 (41.5)	5 (33.3)	15 (36.6)	29 (46.8)	
Peritoneum					.436
No	96 (81.4)	14 (93.3)	33 (80.5)	49 (79.0)	
Yes	22 (18.6)	1 (6.7)	8 (19.5)	13 (21.0)	
Distant LAP					.969
No	102 (86.4)	13 (86.7)	35 (85.4)	54 (87.1)	
Yes	16 (13.6)	2 (13.3)	6 (14.6)	8 (12.9)	

Figures are numbers with percentages in parentheses, unless otherwise stated

The Chi-Squared test of independence: categorical variable

The Kruskal-Wallis test is a nonparametric (distribution free) test: continuous variable

*IQR* Interquartile, *CR* Complete response, *PR* Partial response, *SD* Stable disease, *PD* Progressive disease, *NA* Not assessed *ALP* Alkaline phosphatase, *ALT* Alanine aminotransferase, *NLR* Neutrophil to lymphocyte ratio, *MLR* Monocyte to lymphocyte ratio, *PLR* Platelet to lymphocyte ratio, *LAP* Lymphadenopathy, *PTCD* Percutaneous transhepatic cholangiography drainage, *ICCA* Intrahepatic cholangiocarcinoma, *ECCA* Extrahepatic cholangiocarcinoma, *GB* Gallbladder

### Efficacy of chemotherapy with gemcitabine plus cisplatin

Among the patients with evaluable response, one patient achieved CR, 14 achieved PR, 41 achieved SD, and 48 had PD as their best response. Fourteen patients had no response evaluation and the majority of them experienced rapid progression without radiological confirmation. The RR and DCR in the entire cohort were 12.7 and 47.5%, respectively. Among all the evaluable patients, they were 14.4 and 53.8%, respectively. The median PFS and OS were 3.6 months (95% CI: 2.8–4.4 months) and 8.4 months (95% CI: 6.5–10.2 months), respectively.

### Identification of prognostic factors for PFS (Table 2)

In the univariate analysis, primary cancer sites (*p* = 0.011), NLR (*p* = 0.020), MLR (*p* = 0.028), biliary drainage (*p* = 0.047), metastases to lung (*p* < 0.001), metastases to liver (*p* = 0.034), and tumor response (p < 0.001) were significant prognostic factors for PFS.

**Table 2:** Univariate and multivariate analysis of prognostic factors in patients with (PFS)

Parameters	Median (months)	95% CI	*P* value	Hazard ratio	95% CI	*P* value
Age			.821	–		
**≦**65 (*n* = 74)	3.8	2.7–4.9				
> 65 (*n* = 44)	3.3	2.3–4.2				
Gender			.540	–		
Male (*n* = 58)	2.8	2.2–3.4				
Female (*n* = 60)	4.0	2.8–5.3				
ICD-10 cancer site			.011			
C22.1 – ICCA (n = 86)	3.9	2.6–5.2		0.877	0.321–2.397	.799
C23/C24.9 –GB/others (*n* = 18)	2.7	2.4–3.0		1.568	0.510–4.819	.433
C24.0 – ECCA (*n* = 9)	8.0	0.0–24.4		1		
C24.1 – Ampullary (*n* = 5)	3.3	1.7–4.8		1.519	0.378–6.110	.556
Performance status			.260	–		
0/1 (*n* = 102)	3.8	2.7–4.9				
2/3 (*n* = 16)	2.7	1.8–3.7				
NLR			.020			
**≦**7.45 (*n* = 100)	3.8	2.8–4.8		1		
> 7.45 (*n* = 18)	2.4	1.4–3.3		1.982	1.040–3.777	.038
MLR			.028			
**≦**0.28 (*n* = 40)	5.9	4.3–7.4		1		
> 0.28 (*n* = 78)	2.9	2.5–3.3		1.263	0.737–2.162	.396
PLR			.396	–		
**≦**136.4 (*n* = 47)	4.8	2.9–6.8				
> 136.4 (*n* = 71)	3.1	2.7–3.5				
Albumin (g/dL)			.777	–		
**≦**3.5 (*n* = 33)	3.1	2.6–3.6				
> 3.5 (*n* = 72)	3.9	2.8–4.9				
ALT (U/L)			.484	–		
**≦**36 (*n* = 68)	3.2	2.3–4.0				
> 36 (*n* = 49)	4.3	2.8–5.7				
Bilirubin (mg/dL)			.622	–		
**≦**1.3 (*n* = 89)	3.8	2.6–5.0				
> 1.3 (*n* = 28)	3.3	2.4–4.1				
ALP (U/L)			.060	–		
**≦**94 (*n* = 30)	5.9	2.5–9.2				
> 94 (*n* = 82)	2.8	2.4–3.3				
Creatinine (mg/dL)			.612	–		
**≦**1.27 (*n* = 115)	3.6	2.6–4.6				
> 1.27 (n = 3)	2.7	0.0–6.7				
CA19–9 (U/mL)			.263	–		
**≦**37 (*n* = 35)	2.9	2.3–3.4				
> 37 (*n* = 82)	4.3	2.7–5.9				
CEA (ng/mL)			.347	–		
**≦**5 (*n* = 64)	4.3	3.0–5.7				
> 5 (*n* = 54)	3.2	2.7–3.6				
Biliary drainage			.047			
None (*n* = 88)	3.2	2.4–4.0		1.396	0.497–3.921	.527
Internal drainage(n = 8)	7.6	3.2–12.1		1		
PTCD (*n* = 19)	3.4	2.8–4.0		0.711	0.244–2.066	.531
Both (n = 3)	1.3	0.5–2.1		8.710	1.831–41.445	.007
Tumor involvement						
Primary tumor			.081	–		
No (n = 8)	2.6	2.1–3.1				
Yes (*n* = 110)	3.8	2.7–4.8				
Regional LAP			.679	–		
No (*n* = 42)	3.9	2.6–5.1				
Yes (*n* = 76)	3.2	2.3–4.1				
Lung			<.001			
No (*n* = 95)	4.3	3.0–5.6		1		
Yes (*n* = 23)	2.6	2.2–3.1		1.678	0.905–3.112	.101
Bone			.181			
No (*n* = 105)	3.8	2.7–4.8				
Yes (*n* = 13)	3.4	2.3–4.5				
Liver			.034			
No (*n* = 69)	4.3	2.2–6.3		1		
Yes (n = 49)	3.2	2.1–4.2		1.232	0.759–1.998	.398
Peritoneum			.138	–		
No (*n* = 96)	3.9	2.5–5.2				
Yes (*n* = 22)	3.1	2.5–3.7				
Distant LAP			.785	–		
No (n = 102)	3.4	2.3–4.5				
Yes (n = 16)	3.6	2.5–4.7				
Tumor Response			<.0001			
CR/RR (*n* = 15)	14.1	7.9–20.2		1		
SD (*n* = 41)	7.6	6.4–8.8		1.819	0.824–4.015	.139
PD (*n* = 48)	2.5	2.4–2.7		55.556	19.467–158.550	<.0001
N/A (*n* = 14)	1.3	0.8–1.9		63.905	20.396–200.232	<.0001

*CI* Confidence interval, *CR* Complete response, *PR* Partial response, *SD* Stable disease, *PD* Progressive disease, *N/A* Not assessed, *ALP* Alkaline phosphatase, *ALT* Alanine aminotransferase, *NLR* Neutrophil to lymphocyte ratio, *MLR* Monocyte to lymphocyte ratio, *PLR* Platelet to lymphocyte ratio, *LAP* Lymphadenopathy, *PTCD* Percutaneous transhepatic cholangiography drainage, *ICCA* Intrahepatic cholangiocarcinoma, *ECCA* Extrahepatic cholangiocarcinoma, *GB* Gallbladder

**Fig. 1:**
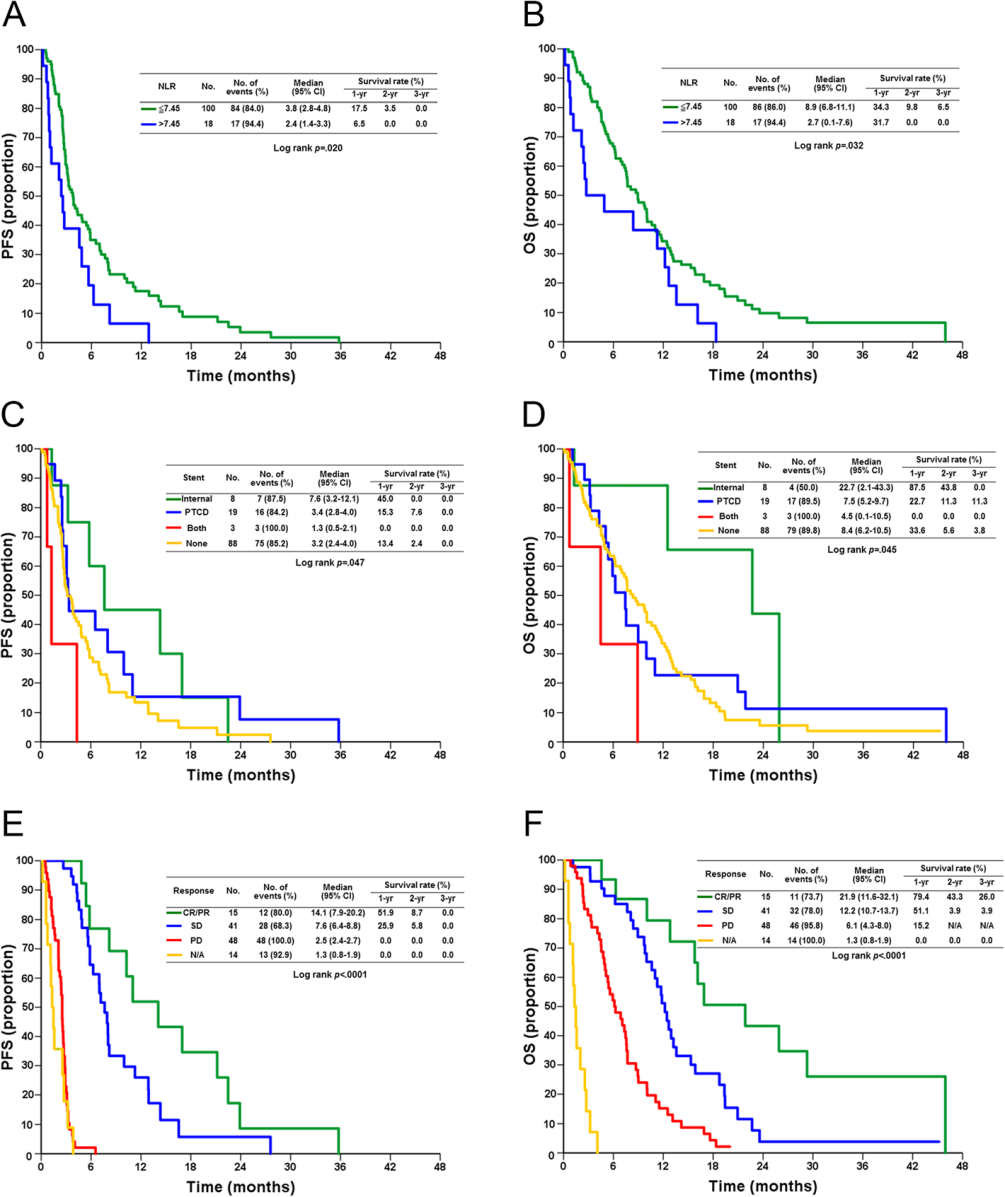
The Kaplan-Meier survival curves of PFS (**a**, **c**, **e**) and OS (**b**, **d**, **f**) for patients, stratified according to independent prognostic factors, NLR (A, B), biliary drainage (**c**, **d**) and tumor responses (**e**, **f**). PFS, progression-free survival; OS, overall survival; NLR, neutrophil to lymphocyte ratio; PTCD, percutaneous transhepatic cholangiography drainage; CR, complete response; PD, partial response; SD, stable disease, PD progressive disease; N/A, not assessed

In the multivariate analysis, NLR > 7.45 (vs. NLR ≤ 7.45, HR: 1.982, 95% CI: 1.040–3.777, *p* = 0.038) (Fig. 1a), biliary drainage requiring both percutaneous transhepatic cholangiography drainage (PTCD) and internal stenting (vs. internal drainage, HR: 8.710, 95% CI: 1.831–41.445, *p* = 0.007) (Fig. 1c), and tumor responses with PD (vs. CR/PR, HR: 55.556, 95% CI: 19.467–158.550, *p* < 0.0001) and N/A (vs. CR/PR, HR: 63.905, 95% CI: 20.396–200.232, p < 0.0001) (Fig. 1e) were independent poor prognostic factors for PFS.

### Identification of prognostic factors for OS (Table 3)

In the univariate analysis, sex (*p* = 0.028), NLR (*p* = 0.032), MLR (*p* = 0.005), ALP (*p* = 0.007), biliary drainage (*p* = 0.045), metastases to lung (*p* = 0.009), metastases to peritoneum (p = 0.032), and tumor response (*p* < 0.001) were significant prognostic factors for OS.

**Table 3:** Univariate and multivariate analysis of prognostic factors in patients with (OS)

Parameters	Median (months)	95% CI	P value	Hazard ratio	95% CI	P value
Age			.285	–		
**≦**65 (*n* = 74)	10.0	8.0–12.0				
> 65 (*n* = 44)	6.9	5.4–8.3				
Gender			.028			
Male (*n* = 58)	6.1	3.6–8.6		1.782	1.151–2.759	.010
Female (*n* = 60)	11.0	9.1–12.9		1		
ICD-10 cancer site			.143	–		
C22.1 – ICCA (*n* = 86)	8.7	6.6–10.9				
C23/C24.9 –GB/others (*n* = 18)	6.1	4.5–7.8				
C24.0 – ECCA (*n* = 9)	10.0	3.3–16.7				
C24.1 – Ampullary (*n* = 5)	12.5	8.5–16.6				
Performance status			.006			
0/1 (*n* = 102)	9.0	7.2–10.8		1		
2/3 (*n* = 16)	3.2	0.0–7.6		1.089	0.568–2.084	.798
NLR			.032			
**≦**7.45 (*n* = 100)	8.9	6.8–11.1		1		
> 7.45 (*n* = 18)	2.7	0.0–7.6		1.922	1.009–3.663	.047
MLR			.005			
**≦**0.28 (*n* = 40)	12.5	11.1–13.9		1		
> 0.28 (*n* = 78)	5.9	3.6–8.3		1.359	0.793–2.328	.264
PLR			.839	–		
**≦**136.4 (*n* = 47)	9.8	7.4–12.1				
> 136.4 (*n* = 71)	7.7	6.1–9.3				
Albumin (g/dL)			.244	–		
**≦**3.5 (*n* = 33)	5.9	2.0–9.8				
> 3.5 (*n* = 72)	9.7	6.8–12.6				
ALT (U/L)			.819	–		
**≦**36 (*n* = 68)	8.1	6.7–9.8				
> 36 (*n* = 49)	9.0	5.7–12.3				
Bilirubin (mg/dL)			.696	–		
**≦**1.3 (*n* = 89)	9.7	7.7–11.7				
> 1.3 (*n* = 28)	7.5	5.6–9.3				
ALP (U/L)			.007			
**≦**94 (*n* = 30)	13.1	10.2–16.1		1		
> 94 (*n* = 82)	6.3	4.2–8.3		2.523	1.470–4.331	.001
Creatinine (mg/dL)			.244	–		
**≦**1.27 (*n* = 115)	8.7	6.9–10.5				
> 1.27 (*n* = 3)	3.2	0.0–8.0				
CA19–9 (U/mL)			.206	–		
**≦**37 (*n* = 35)	6.3	4.5–8.0				
> 37 (*n* = 82)	9.7	7.3–12.1				
CEA (ng/mL)			.358	–		
**≦**5 (*n* = 64)	9.0	6.4–11.6				
> 5 (*n* = 54)	7.7	5.9–9.5				
Biliary drainage			.045			
None (*n* = 88)	8.4	6.2–10.5		2.041	0.708–5.883	.187
Internal drainage (*n* = 8)	22.7	2.1–43.3		1		
PTCD (*n* = 19)	7.5	5.2–9.7		1.559	0.500–4.861	.444
Both (*n* = 3)	4.5	0.0–10.5		6.024	1.253–28.969	.025
Tumor involvement						
Primary			.612	–		
No (*n* = 8)	10.1	7.5–12.6				
Yes (*n* = 100)	8.1	6.2–10.0				
Regional LAP			.265	–		
No (*n* = 42)	11.7	6.2–17.3				
Yes (*n* = 76)	7.7	6.3–9.1				
Lung			.009			
No (*n* = 95)	10.0	8.1–11.9		1		
Yes (*n* = 23)	6.3	3.3–9.2		0.681	0.367–1.263	.223
Bone			.330	–		
No (*n* = 105)	8.9	6.8–11.1				
Yes (*n* = 13)	5.1	3.7–6.5				
Liver			.246	–		
No (*n* = 69)	9.0	7.2–10.8				
Yes (*n* = 49)	7.7	4.6–10.7				
Peritoneum			.032			
No (*n* = 96)	8.9	6.2–11.7		1		
Yes (*n* = 22)	5.9	0.3–11.5		1.712	0.996–2.944	.052
Distant LAP			.408	–		
No (*n* = 102)	8.9	6.7–11.2				
Yes (*n* = 16)	6.3	3.0–9.6				
Tumor Response			<.0001			
CR/RR (*n* = 15)	21.9	11.6–32.2		1		
SD (*n* = 41)	12.2	10.7–13.7		2.430	1.012–5.838	.047
PD (*n* = 48)	6.1	4.3–8.0		10.994	4.397–27.489	<.0001
N/A (*n* = 14)	1.3	0.8–1.9		109.903	33.541–360.113	<.0001

*CI* Confidence interval, *CR* Complete response, *PR* Partial response, *SD* Stable disease, *PD* Progressive disease, *N/A* Not assessed, *ALP* Alkaline phosphatase, *ALT* Alanine aminotransferase, *NLR* Neutrophil to lymphocyte ratio, *MLR* Monocyte to lymphocyte ratio, *PLR* Platelet to lymphocyte ratio, *LAP* Lymphadenopathy, *PTCD* Percutaneous transhepatic cholangiography drainage, *ICCA* Intrahepatic cholangiocarcinoma, *ECCA* Extrahepatic cholangiocarcinoma, *GB* Gallbladder

In the multivariate analysis, male sex (vs. female, HR: 1.782, 95% CI: 1.151–2.759, *p* = 0.010), NLR > 7.45 (vs. NLR ≤ 7.45, HR: 1.922, CI: 1.009–3.663, *p* = 0.047) (Fig. 1b), ALP > 94 U/L (vs. ALP ≤ 94 U/L, HR: 2.523, 95% CI: 1.470–4.331, *p* = 0.001), biliary drainage requiring both PTCD and internal stenting (vs. internal drainage, HR: 6.024, 95% CI: 1.253–28.969, *p* = 0.025) (Fig. 1d), tumor responses with SD (vs. CR/PR, HR: 2.430, 95% CI: 1.012–5.838, *p* = .047), tumor responses with PD (vs. CR/PR, HR: 10.994, 95% CI: 4.397–27.489, *p* < .0001), and tumor responses with N/A (vs. CR/PR, HR: 109.903, 95% CI: 33.541–360.113, p < .0001) (Fig. 1f) were independent poor prognostic factors for OS.

### Identification of predictive factors for response

Since tumor response was the most significant prognostic factor for PFS and OS, we opted to find the possible predictive factors for the tumor response (Table 1). MLR ≤ 0.28 was the only significant predictive factor for the tumor responses (*p* = 0.007). In addition, the patients with CR/PR had significantly lower MLR than the patients with other tumor responses (*p* = 0.043).

Elevated ALP was associated with poor response to gemcitabine and cisplatin. However, this association did not reach statistical significance (*p* = 0.061). In terms of the association between tumour involvement and tumor response, lung metastases showed a non-significant association with tumor response (*p* = .069). None of the patients with lung metastases experienced clinical response in the current study. The RR and DCR in lung-metastatic cases were 0 and 30.4%, respectively. Among non-lung-metastatic cases, they were 15.8 and 51.6%, respectively. All the patients who achieved clinical response, had primary tumours. In other words, the patients who had recurrences after the curative operation, suffered from poor clinical response to first-line chemotherapy with gemcitabine and cisplatin.

## Discussion

In the present study, we retrospectively reviewed 118 patients with advanced BTC undergoing chemotherapy with gemcitabine and cisplatin as first-line treatment. The RR, DCR, median PFS, and OS were 12.7, 47.5%, 3.6 months, and 8.4 months, respectively in the entire cohort. Tumor response, NLR, and biliary drainage requiring both PTCD and internal stenting were the common independent prognostic factors for both PFS and OS. In addition, MLR ≤ 0.28 was the only significant predictive factor for the tumor response.

The clinical outcomes of advanced BTC patients undergoing chemotherapy in current study were not as good as previous clinical trials [5, 17]. Besides the difference of patients’ recruitment between clinical trials and retrospective study, a major reason may be the proportion of the cancer sites. In current study, majority of patients (*n* = 86, 72.9%) patients had iCCA which was higher than in ABC-02 and BT-22 trials, and iCCA was considered a poor prognostic factor in BTC [18, 19].

Previous studies have addressed the prognostic factors in patients with advanced BTC undergoing chemotherapy. Park et al. retrospectively analysed the prognostic factors for OS in patients from prospective phase II or retrospective studies. They identified metastatic BTC, iCCA, liver metastases, ECOG performance status, and ALP as independent prognostic factors [19]. The patients in the aforementioned study received TS-1, gemcitabine/capecitabine, or capecitabine/cisplatin, which is not the standard of care currently. However, these prognostic factors might not be limited to such regimens, as some of the prognostic factors were validated in the subsequent studies.

Other studies have evaluated the prognostic factors for advanced BTC treated with gemcitabine and cisplatin as first-line treatment. The results were similar to the current study. Peixoto et al. retrospectively analysed 106 patients and found that poor ECOG performance status was the only significant unfavourable prognostic factor for OS. In addition, the location of the primary tumour and the sites of advanced BTC were the suggested prognostic factors, although they did not achieve statistical significance [20]. Ishimoto et al. reported 77 patients with pure iCCA and observed that lactate dehydrogenase (LDH), C-reactive protein (CRP), and CEA levels were significantly associated with OS in the multivariate analysis [21]. Suzuki et al. analysed 307 patients and identified poor ECOG performance status, elevated serum LDH, and elevated NLR as independent unfavourable prognostic factors [22]. Salati et al. illustrated NLR, ECOG performance status, CA19–9 and the prognostic nutritional index (PNI), an indicator derived from serum albumin and peripheral lymphocyte count, were prognostic factors for OS in patients undergoing first-line chemotherapy of platinum/gemcitabine combination [23].

In the ABC-02 trial, patients with BTC received either gemcitabine alone or gemcitabine and cisplatin as first-line chemotherapy. In addition to the combined gemcitabine/cisplatin regimen, metastatic disease and ECOG performance status were prognostic factors after the univariate analysis [24]. Derived neutrophil lymphocyte ratio (dNLR) was calculated by the formula absolute neutrophil count/(white blood cell count/absolute neutrophil count). It had a prognostic value similar to NLR [25]. High dNLR was associated with shorter PFS and OS in the retrospective analysis in a cohort from the ABC-02 and the BT-22 studies [26].

All of these studies merely found the possible prognostic factors for OS, but none of them reported the prognostic factors for PFS. The correlation of tumor responses with survival has been seldom evaluated in previous studies of advanced BTC, which were the most important prognostic factors in the current study. Takahara et al. [27] and Neuzillet et al. [28] found that PD for first-line chemotherapy was associated with residual OS after first-line chemotherapy in patients undergoing a second-line chemotherapy. It should be acknowledged that tumor response cannot be an a priori criterium to predict survivals, so that its usefulness is limited in the first-line setting.

Performance status was the most common independent prognostic factor in the previous studies. In the present study, poor ECOG performance score (score > 1) was associated with shorter OS (3.2 vs. 9.0 months, *p* = 0.006) in the univariate analysis but not in the multivariate analysis (*p* = 0.798, HR: 1.089). This finding may have resulted probably from the interaction with other confounding variables and low proportion of patients with ECOG performance status score > 1 (13.6%).

In contrast to the previous reports, we identified pre-treatment NLR > 7.45, obstructive jaundice requiring both PTCD and internal stenting, and no clinical response as the unfavourable factors. Chronic inflammation was reported to play an important role in the development and progression of BTC. NLR or dNLR are inexpensive markers reflecting the host inflammation and were validated in the current and the previous studies [22, 26, 29].

Biliary drainage requiring both PTCD and internal stenting was the only independent prognostic factor for both PFS and OS. In other words, PTCD or stenting alone did not influence the survival outcomes if adequate drainage was achieved with acceptable bilirubin levels. Patients requiring both PTCD and internal drainage might have more complicated diseases than other patients with BTC. Moreover, repeated biliary tract infection would compromise and influence the efficacy of the chemotherapy [15]. This should be interpreted cautiously since only 3 patients out of 118 were subject to both procedures, therefore, the finding appears less meaningful in only a limited minority of patients.

We also analysed the association between disease involvement and clinical outcomes. A specific metastatic organ involvement that is prognostic is still undemonstrated in most of the existing literature. In the univariate analysis, metastases to lung or liver were significant prognostic factors for PFS and metastases to lung or peritoneum were significant prognostic factors for OS. The trends for significance were retained on multivariate analysis by lung and peritoneum metastatic involvement in negatively predicting PFS and OS, respectively. Other than lung involvement was previously described as impacting on OS (liver metastasis in first-line [19] and peritoneal involvement in second-line [28]) but lung metastasis was firstly described in current study. The tumour extension and involvement in advanced BTC reflected the tumour heterogeneity, which might influence the efficacy of cytotoxic chemotherapy.

The present retrospective analysis has some limitations. The retrospective nature of a study always involves biases. The present study was conducted not to investigate the efficacy of the chemotherapy, but to identify the possible prognostic and predictive factors in the real-world practice and to adjust the confounding factors by the multivariate analysis to avoid possible biases. Not all the data were available for all the patients in the current study for comprehensive analysis due to the retrospective nature of the study. Of note that most of the variables evaluated in current study were present for either all or all-but-one patients, with only albumin being present in less than 110 patients. We did not include some factors such as LDH and CRP reported by the previous studies. These factors were not reliable when patients experienced biliary tract infection, which happened commonly in the present study. Furthermore, these patients were treated in a high-volume tertiary-care single institute, which could not fully capture real-world practice in small, peripheral clinics. However, the homogeneity of standard treatment in such a single cancer center could attenuate the weight of confounding factors, which might explain the lack of significance of ECOG performance status.

## Conclusion

We identified three important prognostic factors, namely tumor response, NLR, and biliary drainage for both PFS and OS. MLR was the only significant predictive factor for the tumor response. These findings could provide the physicians with more information to justify the clinical outcomes in patients with advanced BTC in real-world practice.

## Data Availability

The datasets generated AND analysed during the current study are not publicly available due to IRB regulation but are available from the corresponding author on reasonable request.

## Abbreviations

ALP: Alanine aminotransferase, creatinine
BTCs: Biliary tract cancers
CA19–9: Carbohydrate antigen 19–9
CEA: Carcinoembryonic antigen
CR: Complete response
iCCA: intrahepatic cholangiocarcinoma
ECOG: Eastern Cooperative Oncology Group
MLR: Monocyte to lymphocyte ratio
N/A: Not assessed
NLR: Neutrophil to lymphocyte ratio
OS: Overall survival
PD: Partial response
PFS: Progression-free survival
PTCD: Percutaneous transhepatic cholangiography drainage
PD: Progressive disease
SD: Stable disease
